# Oxygen Uptake Prediction for Timely Construction Worker Fatigue Monitoring Through Wearable Sensing Data Fusion

**DOI:** 10.3390/s25103204

**Published:** 2025-05-20

**Authors:** Srikanth Sagar Bangaru, Chao Wang, Fereydoun Aghazadeh, Shashank Muley, Sueed Willoughby

**Affiliations:** 1 Inncircles Technologies Inc., Baton Rouge, LA 70810, USA; srikanth.bangaru@inncircles.com; 2 Bert S. Turner Department of Construction Management, Louisiana State University, Baton Rouge, LA 70803, USA; wsueed1@lsu.edu; 3 Department of Mechanical and Industrial Engineering, Louisiana State University, Baton Rouge, LA 70803, USA; aghazadeh@lsu.edu; 4 Performance Contractors Inc., Baton Rouge, LA 70809, USA; smuley2@outlook.com

**Keywords:** oxygen uptake, wearable sensors, electromyography, inertial measurement unit, machine learning, construction worker safety, occupational risks

## Abstract

The physical workload evaluation of construction activities will help to prevent excess physical fatigue or overexertion. The workload determination involves measuring physiological responses such as oxygen uptake (VO_2_) while performing the work. The objective of this study is to develop a procedure for automatic oxygen uptake prediction using the worker’s forearm muscle activity and motion data. The fused IMU and EMG data were analyzed to build a bidirectional long-short-term memory (BiLSTM) model to predict VO_2_. The results show a strong correlation between the IMU and EMG features and oxygen uptake (R = 0.90, RMSE = 1.257 mL/kg/min). Moreover, measured (9.18 ± 1.97 mL/kg/min) and predicted (9.22 ± 0.09 mL/kg/min) average oxygen consumption to build one scaffold unit are significantly the same. This study concludes that the fusion of IMU and EMG features resulted in high model performance compared to IMU and EMG alone. The results can facilitate the continuous monitoring of the physiological status of construction workers and early detection of any potential occupational risks.

## 1. Introduction

The construction sector relies heavily on manual labor and repetitive activities, often causing significant physical exhaustion among workers. In the U.S., nearly 40% of construction workers report severe fatigue, which can impair decision-making, raise injury risks, reduce efficiency, and compromise work standards [1,2]. Additionally, extreme fatigue—stemming from harsh work environmesnts, extended shifts, and demanding tasks—can worsen these negative outcomes, contributing to musculoskeletal disorders (WMSDs) and declines in productivity [3,4]. Fatigue also hinders both physical and mental performance [5] and has been linked to slip-and-fall accidents, one of the top four deadly hazards in construction, as noted by OSHA [6]. Ref. [7] safety report revealed that construction firms spend approximately USD 189.81 million per week on serious, non-fatal workplace injuries.

To prevent or reduce the physical fatigue or physiological demands associated with construction activities, it is possible to monitor the physiological effort at which workers perform the construction activity and evaluate whether the effort exceeds physiological standards [1,8]. The physical demand or physiological workload of construction work can be determined by measuring the average oxygen uptake (or energy expenditure) or the heart rate while performing the construction activity [1,8]. To assess the physical demands of construction activities, early efforts were made by Abdelhamid and Everett [1] to measure the physiological demands such as heart rate and oxygen uptake using the KB1-C metabolic system, which was cumbersome and uncomfortable.

Photoplethysmogram (PPG) and Electrodermal activity are non-invasive techniques that are commonly used in the field of psychophysiology but are being adopted in other areas, such as construction. The PPG operates based on the optical and transmission properties of the human body at a particular wavelength of light to determine the changes in blood volume in the skin’s microvascular bed [9]. The sensor consists of a light-emitting diode (LED) and a photodetector that senses the emitted light. Based on the position of the LED and the photodetector, PPG is classified as either transmissive or reflective [10]. The term “electrodermal activity” (EDA) refers to the electrical phenomena that occur in the skin and its associated structures, encompassing both the active and passive electrical properties that can be measured [11]. In simpler terms, EDA describes changes in the skin’s ability to conduct electricity. EDA can be assessed through the application of external current (endosomatic method) or through applying alternating current (exosomatic method); in stress-related research, direct current is used [12]. EDA signals can also be reflective of the intensity of our emotional state; therefore, they can be used to identify psychological or emotional arousal episodes [13]. In recent years, with advancements in wearable sensors and machine learning, various researchers developed objectives to assess the workers’ physical demand using physiological sensors such as heart rate, skin temperature, photoplethysmography (PPG), or electrodermal activity (EDA) [3,8,14,15]. Machine learning algorithms have been adopted to help identify non-trivial and complex patterns from biological signal data captured through wearable sensors [16]. Previous studies investigated the percentage change in heart rate [8], rating of perceived exertion [3], and energy expenditure [15] to classify the construction work performed by the worker into various physical demand levels (low–high). Even though heart rate, skin temperature, PPG, or EDA were proven to be able to monitor the individual physical workload, it is still not sufficient for continuously monitoring the physical demand of workers with different individual characteristics (age, gender, and work experience), task characteristics (complex activities in a short interval of time with varying workloads), or for helping quantify the direct impacts of physical workload of activity on construction safety performance or accidents [17,18,19]. To overcome this limitation, there is a necessity to develop a system that can predict physiological demands using data that are dependent on the task and individual characteristics. Therefore, this study proposes to predict oxygen uptake during construction activities using forearm-based wearable sensors and deep learning.

The continuous monitoring of oxygen uptake (VO_2_) helps to evaluate whether the physical demand of the activity exceeds the physiological standards and determines the worker’s physiological status for early indication of accident potentials. Moreover, the motion and muscle activity data obtained from the wearable sensor while workers are performing the activity are influenced by the activity characteristics, individual characteristics, and work conditions. These data could help overcome the limitations of previous studies. The frequency and quality of data obtained from the armband sensor were proven to be sufficient to recognize the complex construction activities in a short interval of time [19,20]. Furthermore, the continuous oxygen uptake prediction helps in measuring aerobic fatigue threshold (AFT), which is used for workers’ fatigue monitoring, as published by the same authors [21]. According to the fatigue monitoring system, the forearm muscle activity and motion data obtained for the workers’ forearm while performing the work are used for worker activity recognition and oxygen uptake prediction. This study evaluated the use of forearm muscle activity and motion data for construction workers’ activity recognition in the previous study [20]. This study focuses on investigating the feasibility of using forearm muscle activity and motion data for oxygen uptake prediction. Since the muscle activity and motion data are highly dependent on the activity, this method is highly suitable for construction as workers are involved in labor-intensive tasks throughout the day.

The rest of this paper is structured as follows. First, we review the current literature on the application of wearable sensors for VO_2_ prediction. Next, the proposed VO_2_ prediction method and the experimental protocol are introduced, followed by the Results Section that evaluates the performance of the proposed VO_2_ prediction method. In the end, it concludes with discussions of the findings, limitations of the study, and future research directions.

## 2. Literature Review

### 2.1. Wearable Sensing Technology Applications in Construction

Wearable sensing technology has been widely adopted in the construction industry for different applications, especially for construction safety and health. Wearable sensing technology such as inertial measurement unit (IMU), electrocardiography (ECG), and seismocardiography (SCG) have been extensively used in different applications for construction health and safety. IMU sensors are quite common; they are embedded in devices or applied in processes to obtain velocity, orientation, and gravitational force. IMU devices have benefited from advancements in technology with a shift from only accelerometers and gyroscope-based devices to the addition of a magnetometer to improve the reading of the gyroscope [22]. In recent years, kinematic sensors such as inertial measurement units (IMUs) have been successfully implemented in several construction applications to monitor a worker’s body posture, acceleration, and orientation [23,24,25] to prevent musculoskeletal disorders from the detected awkward postures [26,27,28] or identify a fall from a high elevation from the identified sudden body acceleration change [29,30,31]. Electrocardiography is a non-invasive procedure that captures the electrical activity in the heart and circulatory system [32]. The diagnostic tool translates the electrical impulses of the heartbeat into a series of waves [33]. ECG has evolved from the traditional systems consisting of electrodes and electrolyte gel to dry electrodes and more recently capacitively coupled electrodes. Studies commonly integrate the sensor with other wearable sensors for various applications. Altini et al. [34] combined a necklace ECG with an accelerometer to estimate the walking speed of participants, activity recognition, and energy expenditure estimation. Unlike ECG, seismocardiography (SCG) monitors mechanical vibrations caused by the heart at the surface of the chest, including vibrations that are below the range of human hearing [35]. Recent studies have used lightweight low-noise accelerometers to enhance the quality of recorded SCG signals [35] such as smartphone accelerometers [36] and laser Doppler vibrometers [37]. A worker’s cardiac activity can also be monitored by wearable sensors such as ECG, SCG, and PPG, and researchers have been studying how to determine the physical and mental condition of the workers using these sensors to monitor the metrics such as heart rate variability (HRV), inter-beat-intervals (IBIs), pulse-rate variability (PRV), and heart-rate reserve (HRR) derived from heart rate [38,39]. Electromyography (EMG) is a method used to record and assess the electrophysiological signals associated with muscle activity, also known as the myoelectric signal. EMG serves as a vital tool for comprehending the muscle activity of the human body, both in typical and abnormal conditions [40]. The non-invasive technique involves placing the electrodes on the muscles of interest and recording signals based on the muscle activity and is referred to as surface electromyography (sEMG) (Systematic review of textile-based electrodes for long-term and continuous surface electromyography recording). Nimbarte [41] studied assessing the muscle load and forces for the ergonomic assessment using the captured muscle activities from the EMG sensors, and other researchers [1,3,42,43] also pioneered the feasibility of assessing workers’ physical workload and fatigue using the PPG, EDA, ST, or heart rate sensors. For construction safety training, eye-tracking and EEG sensors were also implemented to measure workers’ visual attention and brain activities to help evaluate the training effectiveness and monitor trainees’ mental status for training improvement. Although the current literature has shown the feasibility of using wearable sensors for construction safety and health applications, there still exist some challenges, such as high sensor cost, inability to be used for multiple complex activities, noise and artifacts in field measurements, variability in standards to assess personal safety and health risks, the uncertainty of return of investments, and user resistance for adoption [18].

### 2.2. VO_2_ Prediction Using Wearable Sensors

Recent studies explored VO_2_ prediction model development using wearable sensors and machine learning but mainly limited to activities with light-to-moderate intensity such as treadmill walking [17,44], daily living activities [17,45,46,47], cycling exercise [48], and outdoor walking [17]. These studies used physiological and motion sensors such as an electrocardiogram, seismocardiogram, atmospheric pressure, heart rate, respiratory band, Garmin vector power meter, and accelerometer. Except for Shandhi et al. [17], all the other studies have used multiple sensors on different body parts to capture the input data for VO_2_ prediction models. The use of multiple wearable sensors on construction workers while performing the work is cumbersome and unrealistic considering the location of the sensors. Sensor location largely depends on the mechanism of the sensor and the intended application. Aloqlah et al. [49] placed sensors on the head for gait assessment, and while this was effective in that application, sensor signals may suffer interference with hard hats in a typical construction environment. Zignoli et al. [48] placed the sensor on the foot, which obstructs the mobility and interferes with the worker’s steel toe shoes. Moreover, the VO_2_ prediction models were developed using machine learning algorithms such as Logistic regression [45], XGBoost [17], Random Forest [46], Multilayer Perceptron Neural Network [47], Artificial Neural Network [44], and Recurrent Neural Network [48,50] and features such as absolute acceleration, heart rate, cadence, breathing frequency, and power output. Recurrent Neural Networks (RNNs) such as Long Short-Term Memory (LSTM) have been effective for sequence data and regression [50,51]. Even though these studies have achieved acceptable model performance in predicting oxygen uptake, limitations of these studies still exist, such as only light-to-moderate activities were evaluated, the use of multiple sensors for data acquisition, and the inability to predict VO_2_ for high intensity and complex activities performed in short intervals.

### 2.3. Points of Departure

To overcome the above-mentioned limitations in the current literature, this study explores a new method to predict oxygen uptake for complex construction activities through EMG and IMU data collected from only one low-cost armband-based wearable sensor. A bidirectional long-short-term memory (BiLSTM) established a VO_2_ prediction method using the forearm IMU and EMG data. A series of scaffold-building activities were tested to evaluate the performance of the proposed prediction model. Compared with the existing techniques, the preliminary results show that the proposed method has improved results for complex high-intensity construction activities. In addition, this study evaluates and compares the performance of using different sensor features (i.e., IMU alone, EMG alone, and IMU + EMG) and other RNN models. Finally, this study estimates the average VO_2_ required to build one scaffolding unit model using the proposed model.

## 3. Materials and Methods

### 3.1. Data Collection

#### 3.1.1. Participants

Ten active male university students participated in this study (27 ± 1.70 years, 171.7 ± 4.13 cm, 76.70 ± 8.25 kg). The activity level of the participants was moderate-to-vigorous. All the participants were right-handed, non-smokers, and had no lower-back injuries or musculoskeletal disorders. Before starting the experiment, the study’s objective was demonstrated to the participants, and written informed consent was obtained from all the participants. The experiment protocol consistent with the Declaration of Helsinki was approved by the University’s Institutional Review Board (IRB) (ID: IRBAM-20-0539).

#### 3.1.2. Construction Activity Description

**Figure 1:**
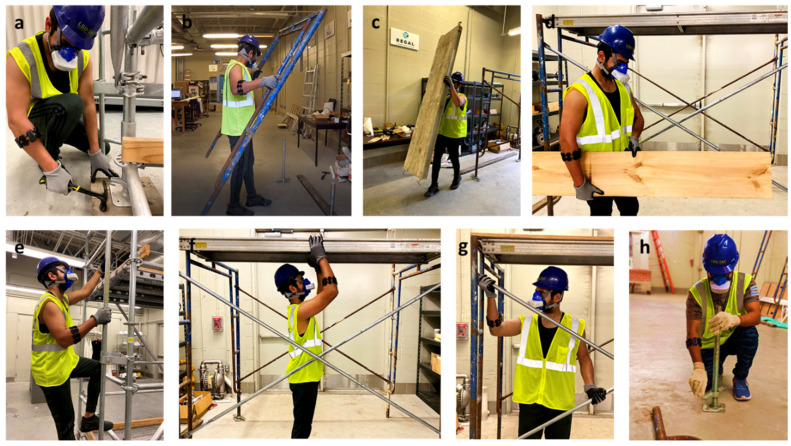
Participants performed different scaffold-building activities. (**a**) Hammering, (**b**) carrying scaffold frame, (**c**) carrying baseboard, (**d**) carrying guardrail, (**e**) going up the vertical ladder, (**f**) installing baseboard on a different level, (**g**) installing crossbars, and (**h**) adjusting leveling jacks.

Construction activities involve heavy labor-intensive tasks and complex motions. In this study, the authors adopted scaffold-building activities that are considered as manual work-intensive activities [20]. One of the significant reasons to choose scaffold-building activities involves moderate-to-heavy workload tasks involving different body part movements (wrist, upper body, forearm, lower body, whole body) and various motions (free motion, repetitive motion, and impulsive motion). This study identified fourteen scaffold-building activities, as shown in Table 1. The tasks involve carrying and installing different objects such as a scaffold frame (38 Lbs.), crossbars (10 Lbs.), leveling jacks (6.5 Lbs.), baseboard (33 Lbs.), and wooden guardrail (5 Lbs.). Also, it involves going up and down the vertical ladder. Figure 1 shows some of the scaffold-building tasks performed by the participant.

#### 3.1.3. Measurements and Instrumentation

**Figure 2:**
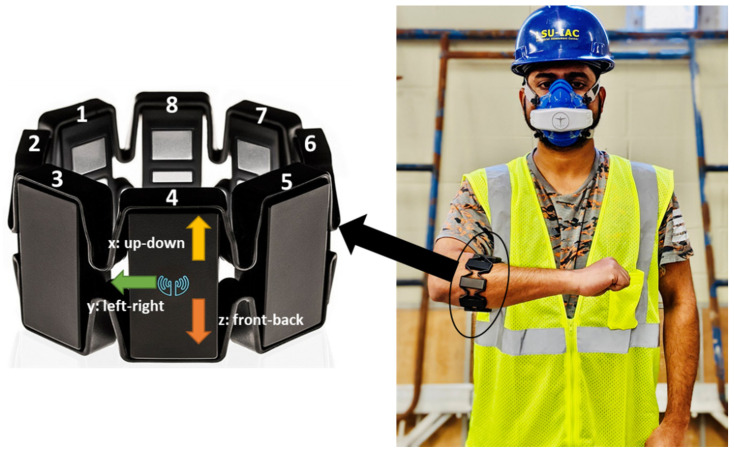
Participant wearing forearm Myo armband sensor and metabolic analyzer.

This study proposes to use a forearm-based wearable armband sensor (Myo Armband) developed by Thalmic Lab Inc. (Kitchener, ON, Canada) to collect forearm IMU and EMG data. The armband consists of eight dry EMG surface electrodes and a 9-axe IMU sensor (3-axe accelerometer, 3-axes gyroscope, and 3-axe magnetometer). The IMU sensor is embedded in the EMG channel 4. The EMG electrodes capture the forearm muscle activity and return an 8-bit array of integer values ranging between −128 and 127, acquired at 200 Hz frequency. In contrast, the IMU data capture the forearm’s motion by measuring acceleration, angular velocity, and orientation in x, y, and z-direction at 50 Hz frequency. The real-time raw IMU and EMG data were transmitted from the armband sensor to local computer storage via Bluetooth Low Energy (BLE) wireless connection. To capture the gold standard breath-by-breath oxygen uptake while performing construction activities, a portable metabolic analyzer, the VO_2_ Master Analyzer (VO_2_ Master Health Sensor Inc., Vernon, British Columbia, CA, USA), was used, as shown in Figure 2. The metabolic analyzer records VO_2_ at a frequency of 1 Hz. The armband sensor and metabolic analyzer were calibrated according to the manufacturer’s guidelines. As shown in Figure 2, the armband is required to be worn at the thickest part of the forearm during the experiment, and the blue Myo logo needs to be located at the lower forearm with the EMG channel 1–4 in the line of the index finger of the participant [21].

#### 3.1.4. Experiment Protocol

Before starting the experiment, participants were asked to warm up their bodies to prevent injuries. Once the participant was ready, the armband sensor and metabolic analyzer were attached to the participant and calibrated using the manufacturer’s guidelines. All the participants performed the fourteen scaffold-building activities for five minutes each. Enough rest was provided between the activities until the heart rate reached below 100 bpm. VO_2_, IMU, and EMG data were recorded continuously for each activity. The activities were performed in a warehouse environment at an average temperature of 72 F. The ten participants’ data were further used for regression model building, training, and evaluation. Moreover, all the participants performed a whole sequence of activities to build one scaffolding unit to simulate the actual work situation. While performing the whole sequence activities, the actual oxygen uptake was measured using the VO_2_ analyzer. The data (unseen dataset) collected while performing the whole sequence were used to determine the average oxygen consumption required to build one scaffold unit.

### 3.2. BiLSTM-Based VO_2_ Prediction

#### 3.2.1. Overview of the Proposed Approach

**Figure 3:**
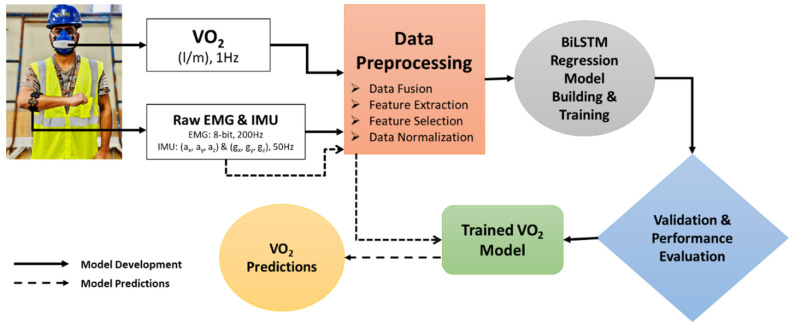
The proposed approach to develop a BiLSTM-based oxygen prediction model using forearm IMU and EMG data.

The proposed framework to develop an oxygen uptake prediction model using forearm-based IMU and EMG data is shown in Figure 3. The raw IMU and EMG data obtained from the armband sensor were preprocessed and synchronized with the oxygen uptake recorded using a VO_2_ analyzer. The preprocessed data were used to train the BiLSTM-based regression model and evaluated using the Leave-One-Subject-Out (LOSO) cross-validation. Furthermore, the trained model was used to predict the oxygen uptake on unseen data to estimate the VO_2_ required to build one scaffold unit.

#### 3.2.2. Data Processing

Since the IMU, EMG, and VO_2_ data were at different frequencies, 17 statistical features were extracted from the raw acceleration, gyroscope, and EMG data for every second without any overlap. Later, the extracted features were synchronized with VO_2_ data at a 1 Hz frequency. In addition to these features, a lag feature was applied by shifting the resultant acceleration (ACC), gyroscope (GYRO), and EMGsum variable and rolling mean for window size by one and mean for window size 3, respectively. Using the lag feature helps the recurrent neural network models see the sufficient past values relevant for future prediction and improves model performance [52]. In total, 289 statistical features and one lag feature were extracted from 17 raw data features, as shown in Table 2. However, all the extracted features may not add value to performance. Additionally, they might cause overfitting problems. Therefore, feature selection techniques such as Pearson’s correlation and mutual information were applied to the 290 features. Pearson’s correlation measures the linear correlation between two features. Simultaneously, mutual information measures the amount of information obtained from one feature given another [53,54]. In this study, the features were selected if Pearson’s correlation and mutual information were more significant than 0.1. Therefore, 69 of 290 features were selected to build the proposed model. The final step of data preprocessing involves data scaling since all the features are in varied scales. For regression and numeric input variables, the normalization technique is used to scale the data between 0 and 1 [55]. After the feature extraction, the dataset of ten participants consists of 52,631 samples with 69 features. The “sample” here refers to a single data point representing one second of synchronized forearm sensor data and corresponding oxygen uptake measurement.

#### 3.2.3. BiLSTM Model Building and Training

**Figure 4:**
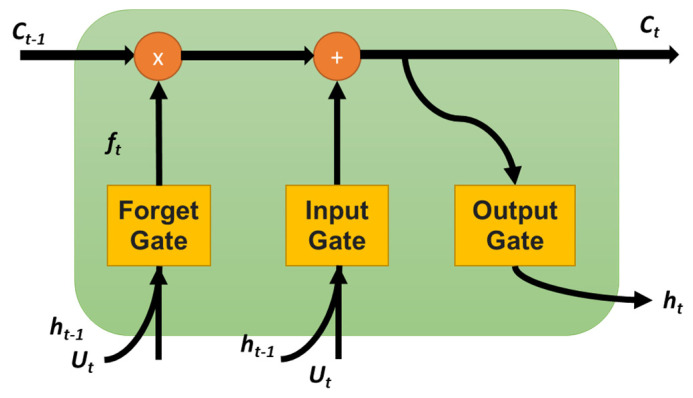
The structure of LSTM cell.

RNN is a type of neural network suitable for sequence data. In RNNs, the recurrent layers store the information from the previous step and combine it with the future timestamp input. Once all the time steps are evaluated, the output layer generates output using the activation function. The output error generated is backpropagated to the network for updating the weights during training and continues until the error is minimized [56]. The most commonly used RNN models for time-series problems are LSTM, BiLSTM, and gated recurrent unit (GRU) [57]. An LSTM cell consists of three gates: input, forget, and output gate, as shown in Figure 4. The input from the previous hidden state and the current input will be sent to these three gates, and the outputs from these are passed to the cell state, which carries the required information. The gates are the neural networks responsible for retaining or removing the information during the training [58]. A BiLSTM-based recurrent neural network is a variation of a long-short-term memory (LSTM) model that consists of a backward and forward LSTM layer to learn information from the past layer [59]. It duplicates the first LSTM layer so that the two layers are trained side-by-side on all the available input data in the past and future timestamps. The combination of backward and forward LSTM layers helps understand the long-term dependencies between the time steps of the sequence data to improve the model performance [60,61,62].

**Figure 5:**
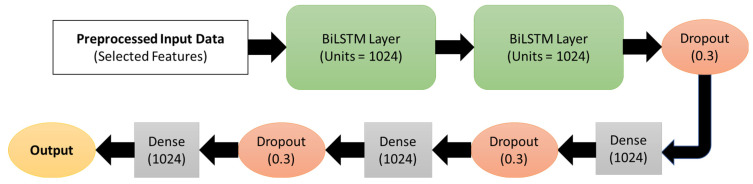
The architecture of the proposed BiLSTM-based oxygen prediction using IMU and EMG.

In this study, the proposed BiLSTM models were implemented in Keras [63], a high-level neural networks API written in Python 3.9.0 and capable of running on top of TensorFlow. The proposed BiLSTM model’s architecture consists of two stacked BiLSTM layers, dropout layers, dense layers, and an output layer, as shown in Figure 5. The data obtained from the preprocessing step were used as input data for the model. The input data consisted of 52,631 samples with 69 features for 10 participants and were reshaped to three-dimensional input for LSTM models, which is in the format of [samples, timestamps, features]. The reshaped input sequence data were fed into two stacked BiLSTM layers with 1024 neurons. The BiLSTM layers’ output was passed through a Dropout (0.3) layer to randomly drop 30% units from the network to prevent overfitting of the model. Later, the data were passed through a series of fully connected dense layers (1024) and a dropout layer (0.3) before reaching the output layer. Since the regression model’s output is a numerical value, no activation function is applied in the final output layer. The hyperparameters such as the number of layers, neurons, optimizers, batch size, and the number of epochs were chosen using the random search hyperparameter optimization technique [64]. An Adam optimizer with a learning rate of 0.001 and a mean square error (MSE) loss function was used to compile the model. An early stopping method with 100 epochs was used to fit the model. The early stopping method helps prevent the model overfit by stopping the training process if there is no improvement in the metrics.

#### 3.2.4. Cross-Validation and Model Evaluation

The leave-one-subject-out cross-validation technique was implemented to evaluate the proposed model’s performance to prevent the overlap between the training and testing datasets, affecting prediction accuracy [65]. Moreover, LOSO helps obtain realistic and generalized model performance on the unseen dataset. In the LOSO method, the dataset of N subjects is divided into N folds or iterations. For each fold, data from (N-1) subjects are used for model training, and the left-out subject data are used for testing. This is repeated with all the N subjects’ data, and the model performance is the average of the results for all subjects. Various metrics such as mean absolute error (MAE), mean square error (MSE), root mean square error (RMSE), coefficient of correlation (R), coefficient of determination (R^2^), and mean absolute percentage error (MAPE) were used to assess the performance of the regression models. MSE is calculated as the squared difference between actual (y) and predicted ($\hat{y}$) output, as shown in Equation (1). MAE measures the magnitude of residuals which is the sum of the absolute difference between actual and predicted outputs, as shown in Equation (2). MSE is the most prominent error, which is calculated using Equation (1). Unlike MAE, MSE is highly sensitive to outliers. The metric RMSE is the standard deviation of the residuals, which helps understand the spread of predicted outputs around expected outputs. RMSE is the square root of MSE, which is calculated using Equation (3). The MSE, MAE, and RMSE are expressed in mL·kg^−1^·min^−1^. This study also reports MAPE, r, and R^2^, which are as shown in Equations (4)–(6), respectively. In addition to evaluating the test dataset, the trained model was implemented on the unseen dataset to determine the error between measured and predicted oxygen uptake.

(1) $$MSE=\frac{1}{n}\sum {\left(y-\hat{y}\right)}^{2}$$

(2) $$MAE=\frac{1}{n}\sum \left|y-\hat{y}\right|$$

(3) $$RMSE=\sqrt{\frac{1}{n}\sum {\left(\hat{y}-y\right)}^{2}}$$

(4) MAPE=100%n∑y−y^y

(5) $$r=\frac{\sum_{i=1}^{n}{\left(\hat{y}-y\right)}^{2}}{\sum_{i=1}^{n}{\left(y-\bar{y}\right)}^{2}}$$

(6) $$R^{2}={\left(\frac{\sum_{i=1}^{n}{\left(\hat{y}-y\right)}^{2}}{\sum_{i=1}^{n}{\left(y-\bar{y}\right)}^{2}}\right)}^{2}$$

## 4. Results

### 4.1. Performances of the Proposed Model

**Figure 6:**
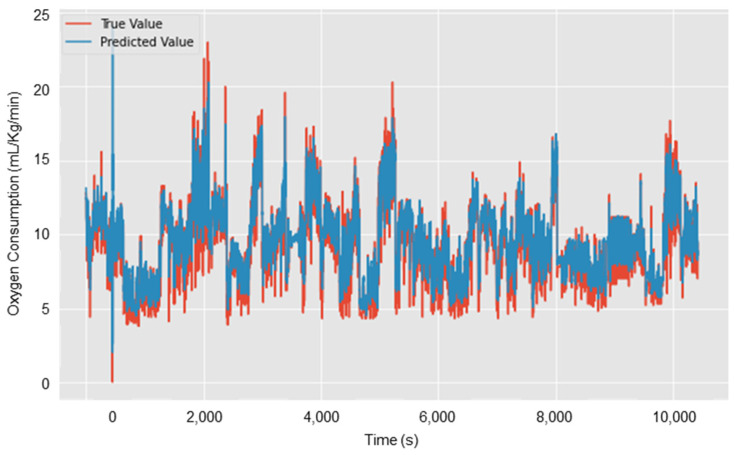
Oxygen uptake prediction on test Subject#1 using the proposed BiLSTM model.

**Figure 7:**
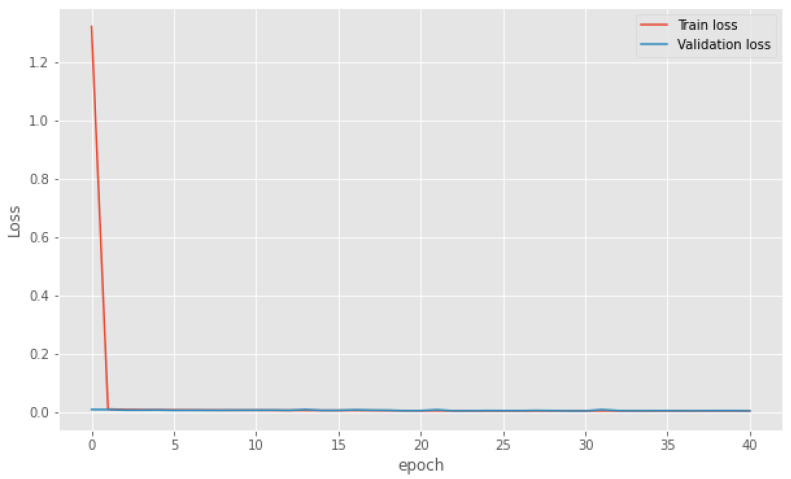
The learning curve of the proposed BiLSTM model using IMU and EMG data.

This section presents the LOSO cross-validation results of the proposed BiLSTM-based regression model using IMU and EMG features. The proposed model was built using 10 participants’ data and 69 selected IMU and EMG features. The average metrics of 10-fold LOSO cross-validation metrics shows are shown in Table 3. The average R^2^, RMSE, and MAE values of the proposed model for all the participants are 0.80, 1.257 mL·kg^−1^·min^−1^, and 1.581 mL·kg^−1^·min^−1^, respectively. Moreover, Table 3 presents the LOSO cross-validation results on test Subject#1, where R^2^, RMSE, and MAE are 0.75, 1.685 mL·kg^−1^·min^−1^, and 1.264 mL·kg^−1^·min^−1^. The measured and predicted oxygen consumption for each second on Subject#1 test data are shown in Figure 6. From Figure 6, it can be observed that the model has predicted the pattern perfectly, but some of the extreme values were missed. Even though the desired results are achieved, it is essential to assess the training performance using learning curves to understand whether the model is suffering from variance or bias [66]. Figure 7 shows that the proposed model loss function (i.e., MSE) decreased with the number of epochs. Moreover, the training and validation loss curves are close to each other, showing that the model has a good fit with low bias and variance.

**Figure 8:**
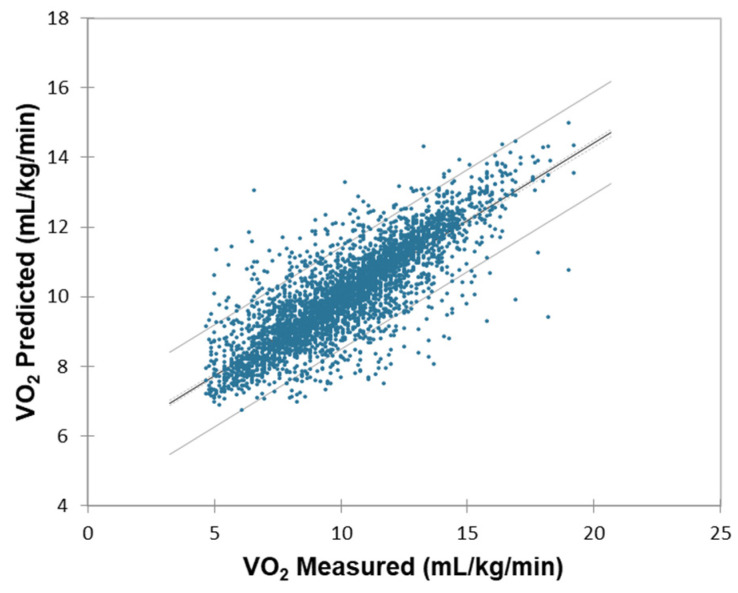
Correlation analysis for measured and predicted VO_2_.

A further trained model was used to predict oxygen consumption on the unseen data (i.e., participants performed an entire sequence of activities to build one unit of scaffold frame). The unseen dataset consists of 4116 number samples from all the participants. The average R^2^, RMSE, and MAE on unseen data of all participants are 0.75, 1.685 mL·kg^−1^·min^−1^, and 1.264 mL·kg^−1^·min^−1^. Figure 8 shows the linear correlation analysis for measured and predicted VO_2_. It should be noted that these results are from second-by-second oxygen uptake predictions.

### 4.2. Average Oxygen Consumption to Build One Scaffolding Unit

The prediction of the proposed BiLSTM model on unseen data of the participants was used to determine the average oxygen consumption required to build a scaffold unit involving fourteen activities shown in Table 1. The average time taken for the participants to build one unit of scaffold is 6.67 minutes (i.e., approximately 400 samples in the unseen dataset for each subject). The average measured and estimated oxygen uptake to build a scaffold unit is 9.18 ± 1.97 and 9.22 ± 1.30, as shown in Table 4. To assess whether the predicted oxygen uptake (VO₂) values were significantly different from the measured values during scaffold-building, we computed the difference column in Table 4 by subtracting the measured VO₂ (in mL/kg/min) from the estimated VO₂ for each participant. To statistically evaluate the agreement between the measured and estimated values, a one-way repeated measures ANOVA was conducted using VO₂ as the dependent variable and condition (measured vs. estimated) as the within-subject factor. The ANOVA results showed no significant difference between measured and estimated VO₂ values across participants (*p* = 0.9641), indicating that the proposed BiLSTM model provides accurate average oxygen consumption estimates for the scaffold-building task. This statistical result supports the model’s potential to serve as a reliable surrogate for direct metabolic measurements in field conditions. It is observed that the error is minimized for the average oxygen consumption over the duration of the build compared to second-by-second predictions. The oxygen consumption estimated using the proposed model can be used to determine the activity’s physical workload. However, it should be noted that the physical workload or oxygen consumption increases with an increase in the work duration and the number of units. 

### 4.3. Comparison with Other RNN Models and Different Sensor Combinations

Furthermore, this study compared other RNN models’ performance and different sensor combinations using the proposed framework (i.e., feature extraction, feature selection, model training, hyperparameter tuning, and LOSO CV evaluation). Three commonly used RNN models (i.e., LSTM, BiLSTM, and GRU) were built for three different sensor feature combinations (i.e., IMU + EMG, IMU alone, and EMG alone). The LOSO cross-validation results for all the models are shown in Table 5. The BiLSTM model has the lowest error and highest correlation for each sensor combination compared to LSTM and GRU. Similarly, the IMU + EMG sensor combination performed better compared to IMU and EMG alone. This shows that the fusion of IMU and EMG features improved the performance of the model.

## 5. Discussions

The results show that the combination of motion (IMU) and muscle activity (EMG) has achieved the highest performance compared to IMU and EMG alone. This is because the fusion of motion and muscle intensity data provides distinctive feature patterns for the model to learn quickly. For example, carrying a guardrail (5 Lbs.) and baseboard (33 Lbs.) can have the same motion, but the muscle intensities are different, helping to detect the oxygen uptake levels. It was previously proved that handling different weights results in different EMG signal patterns [67]. Similarly, the muscle activity is the same for carrying the scaffold frame (38 Lbs.) and baseboard (33 Lbs.), but the motion pattern is different. Since the motion and muscle intensity pattern changes with the physical activity level, the IMU + EMG model performance is higher than others. As suggested by previous studies, sensor data fusion improved model performance compared to individual models [18,20,47,65].

Table 6 compares the current study with similar studies related to oxygen uptake predictions using wearable sensors and machine learning algorithms. For construction applications, it is essential to use a minimum number of sensors to prevent ongoing work obstruction. In the current study, low-cost wearable armband sensors were used to collect forearm IMU and EMG data that can be worn for the entire workday without any discomfort or obstruction to work [20,68]. Except for the study conducted by Shandhi et al. [17], all the previous studies used multiple sensors for data acquisition. However, Shandhi et al. [17] evaluated oxygen consumption only for treadmill walking and the chest strap electrocardiogram sensor might require gel for signal conductivity, which is impractical on construction sites [69]. Moreover, most previous studies have investigated oxygen consumption for daily living activities, walking, and cycling, in light-to-moderate intensity. This is the first study to investigate oxygen consumption using wearable sensors for construction activities. Due to the dynamic nature of the construction activities, different intensity activities are performed in a short interval of time [4]. The use of forearm muscle activity and motion data in this study helped capture the complex movements performed quickly.

Table 7 compares the current and previous studies’ machine learning model performance related to oxygen consumption using wearable sensors. The application of BiLSTM for oxygen uptake prediction is a novel aspect of the current study. The BiLSTM cell’s ability to preserve the information from past and future timestamps helped to model complex data [70]. Moreover, the use of BiLSTM and MSE loss function can handle extreme values compared to LSTM and GRU. The coefficient of determination of the proposed model (R^2^ = 0.80) is lower than that of Zignoli et al. [48] (R^2^ = 0.89) and Borror et al. [44] (R^2^ = 0.91). It should be noted that those studies only evaluated cycling and treadmill activities which are less complex. Lu et al.’s [47] model achieved RMSEs of 1.69, 2.36, 1.62, and 3.88 mL/kg/min for the painting, postal delivery, meat cutting, and lifting activities which are much higher compared to the RMSEs of the proposed model (i.e., 1.26 mL/kg/min). From Table 7, it can be observed that, as the complexity of the activities increases, the performance of the previous models goes down. Except for Shandhi et al. [17], previous studies used raw sensor signals as model input data. The feature engineering process proposed in this study helped to achieve improved model performance on unseen data.

Considering the proposed study’s experimental conditions, the average measured and estimated oxygen consumption for building one scaffold unit is 0.77 and 0.79 L/min, which can be classified as moderate work based on the published work severity guidelines [71]. However, continuous physical activity monitoring is required to evaluate the activity’s workload [1]. The statistical analysis has shown that the measured and estimated average oxygen consumption for building one scaffold unit is similar, providing an opportunity to use the forearm-based IMU and EMG sensor instead of an expensive metabolic analyzer.

The accurate oxygen consumption prediction using a low-cost wearable sensor helps evaluate the activities’ physical workload and quantify the direct impacts of physical workload on construction safety and productivity. Additionally, determining physiological demands by measuring oxygen uptake helps design construction activities with ergonomic interventions to prevent musculoskeletal disorders.

## 6. Conclusions

This study concludes that, using forearm IMU and EMG features, the proposed BiLSTM RNN model can predict second-by-second oxygen uptake. The model achieved a coefficient of correlation and RMSE of 0.90 and 1.257 mL/kg/min using the ten participants’ data of scaffold-building activities. The results show that the data fusion of IMU + EMG (R = 0.90) yielded the highest performance compared to IMU alone (R = 0.88) and EMG alone (0.81). Moreover, the average oxygen consumption for building one scaffold unit is estimated to be 0.77 L/min. The main advantages of the proposed system over previous studies are the use of low-cost sensors, complex construction activities with varying intensities in a short interval of time, the use of a fully automated framework, feature engineering process to improve performance on the unseen dataset, and use of BiLSTM and appropriate hyperparameters to handle complex time-series data. The proposed model can be embedded into the wearable sensor for real-time workload assessment and the worker’s physiological status. The continuous monitoring of oxygen uptake helps evaluate the physical workload of various construction activities, the physiological status of the worker, and early detection of the potential hazards on the construction site.

Even though the result of the study validates the feasibility of using forearm motion and muscle activity data for continuous oxygen monitoring, some of the limitations of this study include the short monitoring time (i.e., for building one scaffold unit), only right-handed male participants, and an indoor warehouse environment. Since the proposed framework is independent of human variability and environmental factors, retraining the model with more diverse participant data on real construction sites can help develop production-level models.

The future work includes implementation of the proposed model on the real construction site for the entire workday to monitor oxygen uptake, understand the influence of sensor position on the model performance, investigate the frequency and time domain features for model building and training, extend the proposed framework for other construction trade activities, and implement time-series data augmentation techniques to improve model performance.

## Figures and Tables

**Table 1:** Scaffold-building activities.

SL. No.	Activities
1	Walking
2	Carrying or Positioning Scaffold Frame
3	Carrying Leveling Jacks
4	Inserting and Adjusting Leveling Jacks
5	Carrying Crossbars
6	Installing Crossbars
7	Hammering
8	Wrenching
9	Carrying and Dragging baseboard
10	Installing Baseboard on Different Level
11	Carrying Guardrail
12	Dragging Guardrail
13	Installing Guardrail
14	Going Up and Down Vertical Ladder

**Table 2:** Feature extraction and selection from raw IMU and EMG data.

Features Extracted from Raw Data		
Dataset	Raw Features	Statistical Features
Acceleration	ax, ay, az, ACC	sum, avg, min, max, median, stdev, cv, var, percentiles (5, 10, 25, 75, 90, 95), skew, kurtosis
Gyroscope	gx, gy, gz, GYRO	
EMG	EMG1, EMG2, EMG3, EMG4, EMG4, EMG5, EMG6, EMG7, EMG8, EMGsum	
ACC, GYRO, EMGsum	Lag Feature, Rolling Mean	Shift (1), rolling (window = 3)
Features Selected for Proposed Model		
Acc_mean, EMGsum_lag1, Gyro_mean, az_per50, az_median, az_avg, az_per25, az_sum, az_per75, az_per10, az_per90, az_per95, az_per5, az_max, ax_max, gz_stdev, ax_per95, gz_min, gz_max, ax_stdev, gz_per95, ax_per90, ay_stdev, gz_per5, az_min, gz_per90, gz_var, gx_stdev, gz_per10, gx_max, ax_per75, gx_min, gy_stdev, res_gyro_max, gy_max, ay_var, res_acc_skew, res_gyro_per95, gy_min, res_gyro_avg, res_gyro_sum, ay_min, ax_sum, ax_var, gx_var, res_gyro_median, res_gyro_per50, res_gyro_stdev, res_gyro_per90, gx_per5, gy_per95, gz_per75, az_skew, EMG7_per25, ax_skew, res_acc_max, res_gyro_per75, gy_per5, gx_per95,ax_avg, res_acc_per95, ax_median, ax_per50, EMG8_per25, EMG8_per75, gy_per10, EMGsum_var, res_gyro_per25, gy_per90		

**Table 3:** LOSO cross-validation metrics of proposed BiLSTM model on test and unseen data.

Model	R	R^2^	MAE	MSE	RMSE	MAPE
All Participants Data	0.895	0.800	0.757	1.581	1.257	10%
Subject#1 as Test Data	0.866	0.750	1.264	2.838	1.685	16%
Unseen Data of All Participants	0.833	0.695	1.236	2.525	1.589	13%

**Table 4:** Measured and estimated oxygen uptake to build one scaffold unit using the proposed model.

Participant		Measured VO_2_		Estimated VO_2_		Difference
	Weight (Lbs.)	mL/kg/min	L/min	mL/kg/min	L/min	
Participant—1	75	9.15	0.69	9.33	0.71	−0.18
Participant—2	74.25	7.67	0.81	8.27	0.78	−0.60
Participant—3	73	9.26	0.68	9.19	0.70	0.07
Participant—4	85	8.24	0.96	8.63	0.91	−0.39
Participant—5	77.70	5.95	0.88	7.09	0.83	−1.13
Participant—6	75	9.35	0.84	9.33	0.80	0.02
Participant—7	63	12.80	0.62	11.67	0.62	1.13
Participant—8	93	8.53	0.82	8.70	0.86	−0.17
Participant—9	81	8.90	0.89	9.02	0.85	−0.12
Participant—10	70	11.98	0.67	10.94	0.68	1.04
Average	76.70	9.18	0.79	9.22	0.77	−0.03
SD	8.25	1.97	0.11	1.30	0.09	0.68

**Table 5:** LOSO CV metrics for different recurrent neural networks and sensor combinations.

Sensor Combination	Model	R	R-Square	MAE	MSE	RMSE	MAPE
IMU + EMG	LSTM	0.871	0.760	1.005	1.905	1.380	11%
	BiLSTM	0.895	0.800	0.757	1.581	1.257	10%
	GRU	0.817	0.667	1.299	2.639	1.624	18%
IMU	LSTM	0.776	0.603	1.509	3.143	1.773	21%
	BiLSTM	0.887	0.787	0.936	1.687	1.299	11%
	GRU	0.469	0.220	2.001	6.173	2.485	22%
EMG	LSTM	0.797	0.636	1.252	2.870	1.694	16%
	BiLSTM	0.816	0.667	1.082	2.627	1.621	13%
	GRU	0.793	0.629	1.254	2.922	1.709	14%

**Table 6:** Comparison of activities and sensors of the current and previous studies on oxygen uptake prediction.

Study	Activities	Activity Type	No. of Sensors	Sensor Signals	Sensor Location
The proposed Study	Scaffold Building	Light—Heavy	1	IMU EMG	Forearm
Zignoli et al. [47]	Cycling Exercise	Light—Moderate	2	Heart Rate, Garmin Vector Power Meter	Foot
Shandhi et al. [16]	Treadmill Walking	Light—Moderate	1	Seismocardiogram Electrocardiogram Atmospheric Pressure	Mid-Sternum
Borror et al. [43]	Treadmill	Light—Moderate	2	Heart Rate Garmin Vector Power Meter	Chest Foot
Lu et al. [46]	Office Painting Postal Delivery Meat Cutting Lifting Tasks	Light—Heavy	8	Electrocardiogram Accelerometer	Chest Wrist Thigh
Beltrame et al. [45]	Daily Living Activities Controlled Walking	Light—Moderate	3	Electrocardiogram Accelerometer Respiratory Bands	Chest Hip
Altini et al. [44]	Daily Living Activities	Light—Moderate	4	Electrocardiogram, Accelerometer	Chest

**Table 7:** Comparison of model performance of the current and previous studies on oxygen uptake prediction.

Study	Activities	Model	R^2^	RMSE
The proposed Study	Scaffold Building	BiLSTM	0.80	1.26
Zignoli et al. [47]	Cycling Exercise	LSTM	0.89	N/A
Shandhi et al. [16]	Treadmill Walking	Xgboost	Treadmill—0.77 Outdoor Walk—0.64	Treadmill (3.68 ± 0.98) Outdoor Walk (4.30 ± 1.47)
Borror et al. [43]	Treadmill	ANN	0.91	N/A
Lu et al. [46]	Office Painting Postal Delivery Meat Cutting Lifting Tasks	MLP	N/A	Office—0.86 Painting—1.69 Postal Delivery—2.36 Meat Cutting—1.62 Lifting—3.88
Beltrame et al. [45]	Daily Living Activities Controlled Walking	Random Forest	Daily Living Activities—0.75 Random Walking—0.48	N/A
Altini et al. [44]	Daily Living Activities	Linear, Exponential Logistic	N/A	4.38 ± 0.80

## Data Availability

The data presented in this study are available upon request from the corresponding author.
